# Sensitivity of various body indices and visceral adiposity index in predicting metabolic syndrome among Chinese patients with adult growth hormone deficiency

**DOI:** 10.1007/s40618-017-0621-2

**Published:** 2017-02-23

**Authors:** L. Qing, R. Wei, L. Chan, Z. Xiaoya, X. Xin

**Affiliations:** Department of Endocrinology, Chongqing Medical University First Affiliated Hospital, #1 You-Yi Rd., Yu-zhong District, Chongqing, 400016 China

**Keywords:** Visceral adiposity index, Body adiposity index, Metabolic syndrome, Adult growth hormone deficiency

## Abstract

**Aim:**

Adult growth hormone deficiency (AGHD) refers to decreased secretion of growth hormones in the adults, which is associated with increased clustering of conventional cardiovascular risk factors such as central obesity, insulin resistance and dyslipidemia. Metabolic syndrome (MetS), a recognized risk factor of cardiovascluar diseases, shares some clinical features. Given that the prevalence of MetS is on the rise in patients with AGHD, and that cardiovascular disease (CVD) is an important cause of morbidity and mortality in that population, the alternative, simple, non-invasive methods of assessing MetS among this population are needed. This study aims to determine the sensitivity of five anthropometric indices [Body mass index (BMI), Waist circumference (WC), Waist-to-hip ratio (WHR), Waist-to-height ratio (WHtR) and Visceral adiposity index (VAI)] in predicting metabolic syndrome in Chinese population-based patients with adult growth hormone deficiency.

**Materials and methods:**

A total of 96 Chinese patients with adult growth hormone deficiency were included in this study. They were compared with equal number of apparently healthy persons with similar characteristics (matched with age and gender) to the previous group. Anthropometric measurements including weight, height, serum lipids indices, blood pressure (BP), fasting plasma glucose (FPG), WC were measured. BMI, WHR, WHtR, and VAI were calculated.

**Results and discussion:**

AGHD patients with MetS had higher WC (91.00 ± 8.28 vs 78.01 ± 7.12), BMI (24.95 ± 2.91 VS 23.30 ± 2.80), WHR (0.92 ± 0.06 VS 0.87 ± 0.07), WHtR (0.53 ± 0.06 VS 0.47 ± 0.05), VAI [(5.59 (4.02, 7.55) VS 1.69 (0.87, 3.05)] levels in comparison to those without MetS. Meantime WC, BMI, WHR, WHtR, VAI was positively correlated to MetS components. ROC curve for participants with AGHD showed that VAI had the highest SS of 92% (BMI 0.812; WHR 0.706; WHtR 0.902; VAI 0.920, respectively) for prediction of MetS in AGHD. The optimal cutoff values for different adiposity markers in predicting MetS were as follows: WC (79.65), BMI (23.46); WHR (0.89); WHtR (0.54); VAI (2.29).

**Conclusion:**

In conclusion, our study showed all adiposity measures of interest present themselves as easy and practical tools for use in population studies and clinical practice for evaluating MetS in AGDH and VAI was identified as the best in Chinese AGHD patients among them.

## Introduction

Adult growth hormone deficiency (AGHD) refers to decreased secretion of growth hormones in the adults, which results from diseases of the pituitary gland or from diseases of the hypothalamus. The clinical manifestations of AGHD depend upon the cause as well as the type and degree of hormonal insufficiency. Increased clustering of conventional cardiovascular risk factors such as central obesity, insulin resistance and dyslipidemia has been demonstrated in patients with AGHD [1]. According to the World Health Organization (WHO) or the National Cholesterol Education Program—Third Adult Treatment Panel (ATP III), Metabolic syndrome (MetS) is increasingly recognized as a distinct entity which comprises the following components: central obesity, hyperglycaemia, hypertension and dyslipidaemia [2, 3]. It pointed out that the MetS shares clinical features with adult growth hormone deficiency such as central obesity, insulin resistance and dyslipidemia. One of other features common to both conditions is premature atherosclerosis and increased mortality from cardiovascular diseases [4, 5].

In addition, MetS is highly prevalent in hypopituitary patients with GHD of adult onset, as a prevalence of 43.1% according to the NCEP definition and of 49.1% according to the IDF definition was found in a study of 2479 hypopituitary patients with adult-onset GH deficiency [6]. A study of moderately–severely obese individual with AGHD has addressed the possible role of GH in the occurrence of MetS. In the study, compared with patients of nomorl growth hormone, AGHD patients show a higher prevalence of MetS, suggesting AGHD to be an independent factor in the development of MetS [7].

These data demonstrate the association between the MetS and AGHD, and emphasize the significance of both AGHD and MetS as diseases accompanied with an adverse cardiovascular risk profile. Apparently, appropriate indices in predicting MetS among patients with adult growth hormone deficiency is insufficient to improve this adverse cardiovascular risk profile.

AGHD was associated with the development of visceral obesity [8]. According to several epidemiological studies, visceral obesity is the most predictive fat component of cardiovascular (CV) disease and events, and visceral obesity is a central feature of metabolic syndrome [9]. Recently, the AlkaMeSy Study Group introduced a index, named visceral adiposity index (VAI), which comprises simple anthropometric parameters including body mass index (BMI), waist circumference (WC) and metabolic parameters [triglycerides (TG) and high-density lipoprotein cholesterol (HDL-C)], which was used as a marker of both visceral fat dysfunction and MetS [10]. Besides, the research of Somma et al. shows a strong relationship between GH axis, VAI and metabolic risk in healthy adults [11]. To date, little information is available regarding the relation between VAI and AGHD. The purpose of the present study was to investigate the association between VAI and AGHD, and the sensitivity of VAI and other traditional adiposity measures such as BMI, WC, waist-to-hip ratio (WHR), waist-height ratio (WHtR) in predicting MetS among Chinese patients with AGHD.

## Materials and methods

### Patients

GH secretion of subjects were evaluated by insulin tolerance test, the gold standard for diagnoses of adult GH deficiency [12, 13]. According to the test, subjects with GH peak value <5.0 µg/L were diagnosed as AGHD while GH peak value <3.0 μg/L were diagnosed as severe AGHD. Finally, a total of 96 patients who confirmed diagnosis of AGHD with a duration of at least 2 years, who are recruited from the Department of Endocrinology of First Affiliated Hospital of Chongqing Medical University during February 2009 to October 2014, were enrolled (30 men and 66 women, aged 46.12 ± 11.32 years). Equal number of apparently healthy persons with similar characteristics (matched with age and gender) to the previous group were recruited as control group. The control group was also evaluated by insulin tolerance test to exclude they are AGHD.

Inclusion criteria: none of the patients had ever received GH therapy and all patients had been receiving adequate replacement therapy of the rest of pituitary hormones. Hormone levels but GH of all patients was maintained within the normal reference range.

The exclusion criteria for entering this study for both groups were: (1) current treatments with drugs known to interfere with glucose, lipid metabolism or blood pressure; (2) presence of previous diagnosis and already known diabetes mellitus or hypertension or a history of malignant tumor; (3) already treatment with GH; (4) liver and kidney functional disorders or mental disorder.

### Methods

The present study was approved by the ethics committee of the University and adhered to the tenets of the Declaration of Helsinki. Additionally, the written informed consents were signed by all participants.

All subjects were interviewed by one person. Interview questions collected consisted of smoking status, medical history, and level of physical activity. All subjects were wearing only underwear without shoes when anthropometric measurements were performed. Anthropometric measurements included weight, height, WC, total cholesterol (TC), TG, HDL-c, low-density lipoprotein cholesterol (LDL-c), blood pressure (BP), fasting plasma glucose (FPG). Body weight was measured to an accuracy of ±0.2 kg. Height, WC and hip circumferences (HC) were measured to minimum recorded unit 0.1 cm.

WHR was calculated as waist circumference divided by hip circumference, and WHtR was computed as waist circumference divided by height. BMI formula is weight in kilograms divided by height in meters squared. VAI was calculated according to the definition established by Amato and colleagues [10], men: $\text{VAI}\,=\,\text{WC}/[\text{39}.\text{68 }+\text{ }(\text{1}.\text{88}\,\times \,\text{BMI})]\,\times \,\text{TG}/\text{1}.0\text{3}\,\times \,\text{1}.\text{31}/\text{HDL}$; women: $\text{VAI}\,=\,\text{WC}/[\text{36}.\text{58}+(\text{1}.\text{89}\,\times \,\text{BMI})]\,\times \,\text{TG}/0.\text{81}\,\times \,\text{1}.\text{52}/\text{HDL}$.

The ATP III guidelines state that the MetS may be diagnosed when a person has three or more of five components. These components are: central obesity, importantly, the ATP III definition includes waist circumference as the measure of obesity. (Chinese men: waist circumference >90 cm, Chinese women: waist circumference >80 cm), an elevated TG level [TG ≥1.7 mmol/l (150 mg/dl)], a reduced HDL cholesterol level [men: <1.03 mmol/l (40 mg/dl); women: <1.29 mmol/l (50 mg/dl)], elevated blood pressure (SBP ≥ 130/DBP ≥ 85 mmHg) and an elevated fasting glucose concentration [FPG ≥ 5.6 mmol/l (100 mg/dl)] [3].

### Statistical analysis

Acquired data were analyzed by SPSS19.0 statistical software. The significance level adopted was 1% (*p* < 0.01). Variables were represented by mean, standard deviation, and frequency. The Kolmogorov–Smirnov test was used to assess if the variables followed a normal distribution. Before statistical analysis, not normally distributed parameters were logarithmically transformed to approximate a normal distribution. The Student’s *t* test for independent samples was applied to compare anthropometric and biochemical profiles between groups. Pearson’s Chi-square test was used to compare behavioral characteristics and assess the difference in prevalence of (components) the MS between AGHD and the control group. The Pearson correlation tests were conducted to correlate the analyzed anthropometric, biochemical, and pressure parameters with MetS components. The receiver operating characteristic (ROC) curve was constructed and the area below the curves was calculated with a 95% confidence interval.

## Results

The anthropometric and metabolic parameters of study participants are summarized in Table 1. AGHD reported smoking more frequently than the control while level of physical activity less frequently than the control, but all of that are not significantly different. Age, weight and height were similar between both groups. There was a significant difference between groups for WC, WHR, WHtR, SBP, DBP, TG, HDL-c, FPG, FINS, HOMA-IR, and VAI. Those are significantly higher in AGHD group, while HDL-c was lower in this group (*p* < 0.01).

**Table 1:** Comparison of clinical characteristics in control and AGHD

Variable	AGHD group (*n* = 96)	Control group (*n* = 96)	*p* value
Patients no (M/F)	30/66	30/66	
MetS no. (*N*)	50	30	
Smoking status (%)	32 (33.3)	31 (32.2)	0.878
Leisure time physical activity (%)			
No	40 (41.6)	36 (37.5)	0.679
Yes, <150 min/week	38 (39.5)	41 (42.7)	0.660
Yes, ≥150 min/week	18 (18.7)	19 (19.7)	0.855
Age (year)	46.26 ± 11.26	45.64 ± 13.03	0.723
Height (m)	1.60 ± 0.07	1.59 ± 0.07	0.799
Weight (kg)	59.49 ± 10.73	57.20 ± 9.59	0.104
BMI (kg/m^2^)	23.20 ± 3.38	22.41 ± 2.59	0.072
WC (cm)	85.60 ± 10.14	75.70 ± 9.04	0.000
WHR	0.90 ± 0.07	0.82 ± 0.06	0.000
WHtR	0.53 ± 0.06	0.47 ± 0.05	0.000
SBP (mmHg)	123.81 ± 14.48	115.54 ± 15.23	0.000
DBP (mmHg)	83.30 ± 8.21	75.18 ± 8.29	0.000
TC (mmol/L)	4.76 (4.00, 5.86)	4.66 (4.11, 5.14)	0.000
TG (mmol/L)	1.71 (1.25, 2.57)	1.28 (0.87, 1.94)	0.000
HDL-C (mmol/L)	1.02 (0.83, 1.33)	1.58 (1.31, 2.12)	0.000
LDL-C (mmol/L)	2.99 (2.64, 3.68)	2.37 (2.03, 2.93)	0.000
FPG (mmol/L)	5.50 (4.96, 6.27)	4.68 (4.23, 5.23)	0.000
FINS (mU/ml)	6.43 ± 3.46	4.57 ± 1.80	0.000
HOMA-IR	2.06 (1.97, 2.14)	1.06 (0.81, 1.37)	0.000
VAI	3.47 (1.67, 5.94)	1.05 (0.67, 1.89)	0.000

*MetS* metabolic syndrome, *BMI* body mass index, *WC* waist circumference, *WHR* waist-to-hip ratio, *WHtR* waist-to-height ratio, *VAI* visceral adiposity index, *SBP* systolic blood pressure, *DBP* diastolic blood pressure, *FPG* fasting serum glucose, *TG* triglyceride, *TC* total cholesterol, *HDL-C* high-density lipoprotein cholesterol, *LDL-C* low-density lipoprotein cholesterol, *FINS* fasting insulin, *HOMA-IR*, homeostasis model of assessment for insulin resistence index

**Fig. 1:**
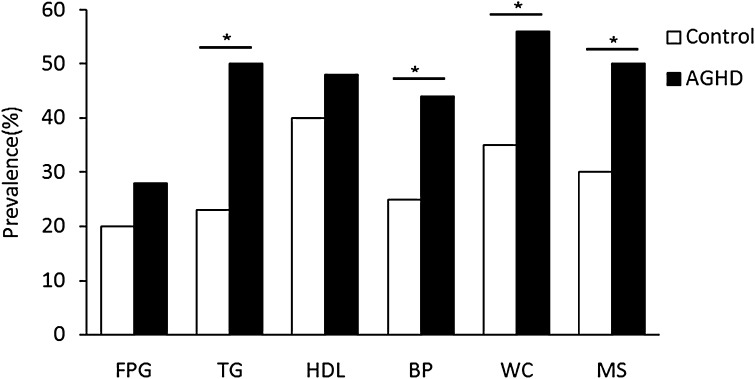
The prevalence of (components of) the metabolic syndrome in patients with growth hormone deficiency at baseline compared with healthy controls

Among 96 AGHD group, 52.1% of AGHD patients fulfilled the definition for MetS in this study, which is higher than the controls (31.3%). Comparison of components of the MetS: hypertriglyceridemia [52.1 vs 24.0%, (*p* < 0.001)], hypertension [45.8 vs 26%, (*p* < 0.001)] and abdominal obesity [58.3 vs 36.5%, (*p* = 0.002)] were significantly more prevalent in patients compared with controls (Fig. 1). Compare anthropometric and biochemical profiles between AGHD with MetS and without MetS shown in Table 2. No differences were observed for age, height, LDL-c between groups. However, in AGHD with MetS group, BMI, WC, WHR, WHtR, VAI, TG, SBP, DBP, VAI, FPG, FINS, HOMA-IR were significantly increased (*p* < 0.01), while HDL-c were significantly decreased (*p* < 0.01).

**Table 2:** Comparison of anthropometric and biochemical profiles between AGHD with MetS and without MetS

Variable	MetS group (*n* = 50)	Non-MetS group (*n* = 46)	*p* value
Age (year)	47.70 ± 10.61	44.70 ± 11.84	0.193
Height (m)	1.60 ± 0.06	1.59 ± 0.07	0.642
Weight (kg)	64.20 ± 10.96	57.90 ± 9.39	0.079
BMI (kg/m^2^)	24.95 ± 2.91	23.30 ± 2.80	0.000
WC (cm)	91.00 ± 8.28	78.01 ± 7.12	0.000
WHR	0.92 ± 0.06	0.87 ± 0.07	0.000
WHtR	0.53 ± 0.06	0.47 ± 0.05	0 000
SBP (mmHg)	128.70 ± 15.10	118.50 ± 11.72	0.000
DBP (mmHg)	87.37 ± 6.94	79.28 ± 7.81	0.000
TC (mmol/L)	4.74 (3.76, 6.00)	4.85 (4.17, 5.72)	0.000
TG (mmol/L)	2.39 (1.76, 2.85)	1.30 (1.65, 1.82)	0.000
HDL-C (mmol/L)	0.88 (0.73, 1.02)	1.29 (1.04, 1.66)	0.000
LDL-C (mmol/L)	3.03 (2.70, 3.78)	2.95 (2.39, 3.28)	0.000
FPG (mmol/L)	6.21 (5.60, 6.81)	5.20 (4.67, 5.50)	0.000
FINS	7.43 ± 4.07	4.19 ± 2.93	0.000
HOMA-IR	1.91 ± 1.09	0.99 ± 0.77	0.000
VAI	5.59 (4.02, 7.55)	1.69 (0.87, 3.05)	0.000

*BMI* body mass index, *WC* waist circumference, *WHR* waist-to-hip ratio, *WHtR* waist-to-height ratio, *VAI* visceral adiposity index, *SBP* systolic blood pressure, *DBP* diastolic blood pressure, *FPG* fasting serum glucose, *TG* triglyceride, *TC* total cholesterol, *HDL-C* high-density lipoprotein cholesterol, *LDL-C* low-density lipoprotein cholesterol, *FINS* fasting insulin, *HOMA-IR* homeostasis model of assessment for insulin resistence index

**Fig. 2:**
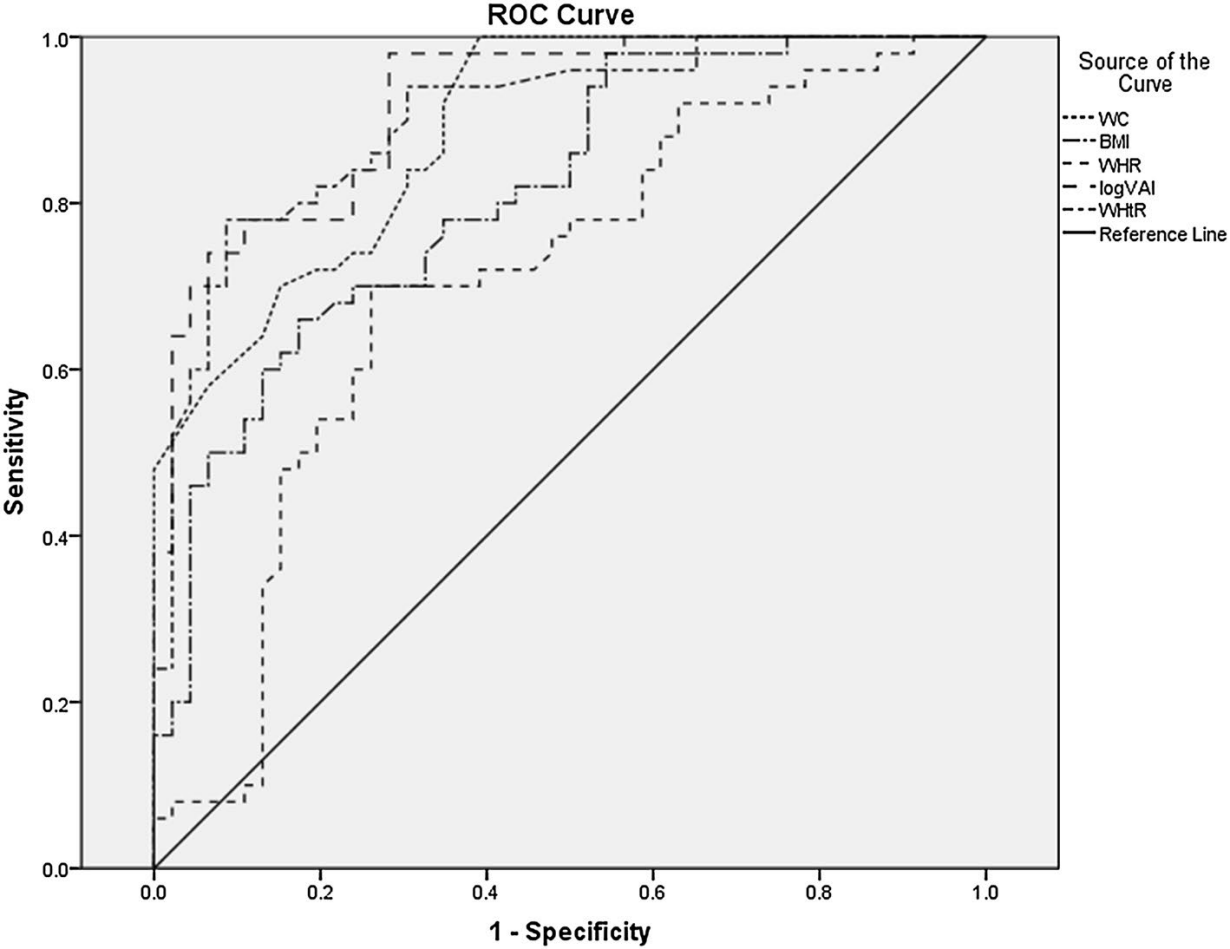
The ROC analysis of adiposity markers in predicting MetS in adult growth hormone deficiency

Results of the analyses of the association between MetS components and insulin resistance with anthropometric adiposity indicesare shown in Table 3. WC, BMI and WHtR showed significant correlation with all MetS components and HOMA-IR among AGHD. There was a strong significant correlation between VAI and all MetS components except SBP. Meanwhile, WHR was only significantly associated with SBP, FPG, HOMA-IR (*p* < 0.01). Somehow, all adiposity measures of interest present themselves associated with MetS components. Further, the ROC curve for participants with AGHD showed the optimal cutoff values for different adiposity markers in predicting MetS as follows: WC: 79.65 cm, BMI: 23.46 kg/m^2^; WHR: 0.89 cm/cm; WHtR: 0.54 cm/cm; VAI: 2.29. Among AGHD, we found that VAI had the highest SS of 92% (BMI: 0.812; WC: 0.888; WHR: 0.706; WHtR: 0.902; VAI: 0.920, respectively) for prediction of MetS in Table 4 and Fig. 2.

**Table 3:** Correlation between components of MetS and various body indices

Variable	BMI (kg/m^2^)	WC (cm)	WHR	WHtR	VAI
SBP (mmHg)	0.444^**^	0.448^**^	0.199^**^	0.460^**^	0.078
DBP (mmHg)	0.607^**^	0.505^**^	0.296	0.451^**^	0.286^**^
TG (mg/dL)	0.517^**^	0.316^**^	0.114	0.341^**^	0.877^**^
HDL-c (mg/dL)	−0.502^**^	−0.429^**^	−0.144	−0.391^**^	−0.395^**^
FPG (mg/dL)	0.671^**^	0.526^**^	0.285^**^	0.515^**^	0.517^**^
HOMA-IR	0.436^**^	0.543^**^	0.430^**^	0.469^**^	0.357^**^

*BMI* body mass index, *WC* waist circumference, *WHR* waist-to-hip ratio, *WHtR* waist-to-height ratio, *VAI* visceral adiposity index, *SBP* systolic blood pressure, *DBP* diastolic blood pressure, *FPG* fasting serum glucose, *TG* triglyceride, *HDL-C* high-density lipoprotein cholesterol, *HOMA-IR* homeostasis model of assessment for insulin resistence index

**p < 0.05

**Table 4:** ROC curves and appropriate cutoff of adiposity indexes in metabolic syndrome prediction in AGHD

Variables	Cutoff	Area	95% Confidence interval	*p* values
BMI (kg/m^2^)	23.45	0.812	(0.728, 0.896)	0.000
WC (cm)	79.65	0.888	(0.827, 0.950)	0.000
WHR	0.89	0.706	(0.599, 0.812)	0.001
WHtR	0.54	0.902	(0.841, 0.962)	0.000
VAI	2.29	0.920	(0.868, 0.971)	0.031

Table 3 shows the correlations between VAI and metabolic variables. Irrespective of gender, VAI was significantly correlated with MetS components except SBP (*p* < 0.05). Then, we further analysis of correlated with MetS components by gender (Table 5). The significant association of VAI with DBP, FPG (*p* < 0.01) in females and DBP, FPG (*p* < 0.05) in males existed. While other significant association of VAI and TG, HDL-C, HOMA-IR existed in both genders (*p* < 0.01). Even after adjusting the age, the association existed.

**Table 5:** Correlation between VAI and metabolic syndrome components

	SBP	DBP	FPG	TG	HDL-C	HOMA-IR
Men						
VAI	0.390	0.495^*^	0.482^*^	0.763^**^	−0.864 ^**^	0.583^**^
VAI	0.382	0.488^*^	0.477^*^	0.765^**^	−0.870^**^	0.595^**^
(Age adjusted)						
Women						
VAI	0.235	0.496^*^	0.463^*^	0.716^**^	−0.569^**^	0.391^**^
VAI	0.215	0.488^*^	0.462^*^	0.715^**^	−0.569^**^	0.384^**^
(Age adjusted )						

*SBP* systolic blood pressure, *DBP* diastolic blood pressure, *FPG* fasting serum glucose, *TG* triglyceride, *HDL-C* high-density lipoprotein cholesterol, *HOMA-IR* homeostasis model of assessment for insulin resistance index

^*^The *P* values listed are less than or equal to 0.005

^**^The *P* values listed are less than or equal to 0.001

## Discussion

Epidemiological studies have demonstrated that hypopituitarism without growth hormone replacement therapy is associated with an increased risk of cardiovascular events, even a high incidence of cardiovascular mortality [14]. So a contributory factor to this has been hypothesized to be GH deficiency [15, 16]. In our study, observations, showing that AGHD patients have many adverse cardiovascular risk profiles, can explain to some extent in the increased cardiovascular mortality of AGHD. One of the observations was dyslipidemia. Comparing with healthy controls, AGHD patients were characterized by increased concentrations of TG, LDL-c, and decreased concentrations of HDL-c. The adverse lipid profiles are in agreement with previous observations shown in AGHD adults [5, 16, 17]. Second, we have observed that AGHD has significantly higher BMI, WC, VAI, WHR, WHtR than controls. BMI is a measure of overall obesity. WC, WHR are used as indicators of abdominal obesity. While VAI and WHtR can take into account adipose tissue distribution [10, 18–20]. Thus, an enlarged WC or VAI could be due to increased abdominal subcutaneous or visceral adipose depots. Although the best adiposity measurement for predicting CVD remains controversial, BMI, WC, VAI, WHR and WHtR were correlated with the cardio-metabolic risk factors [20]. What is more, visceral adiposity itself was a key driver of the cardio-metabolic risk [21]. In a word, visceral obesity may be one risk of increasing cardiovascular events of AGHD. Third, impaired glucose metabolism characterized by insulin resistance and fasting hyperinsulinemia is often highly prevalent in AGHD [7]. In our study, we observed the same result that HOMA-IR, FINS, FPG are higher in AGHD group. Taken together, it has demonstrated that AGHD has high risks of cardiovascular events, and three main reasons could be considered as visceral obesity, adverse lipid profiles and insulin resistance.

The metabolic syndrome—a collection of factors associated with increased risk of cardiovascular disease and diabetes—is becoming increasingly common. Our study has observed that there is a high prevalence of MetS in AGHD. Hypertriglyceridemia, hypertension and abdominal obesity were more prevalent in untreated patients when compared with the age-matched controls, resulting in a higher prevalence of the MetS in patients (52.0 vs 29.1%, respectively). The present findings are consistent with other previous results [22]. Some nonsystematic error in reporting of smoking history, physical activity of two groups has no significant differences, which can have an impact on anthropometric and metabolic parameters. AGHD with MetS have more unfavorable lipid abnormalities, abdominal obesity, and insulin resistance than those of non-MetS, which is increasing cardiovascular disease risks. It is generally thought that intervention for the AGHD with MetS is recombinant human growth hormone (rhGH), but this is insufficient to normalize some risk factors in patients. However, at present there are no approved drugs that can reliably reduce all of the metabolic risk factors over the long term. Studies reported that during rhGH replacement at a mean dose of 0.5 +/− 0.2 mg/day resulting in IGF-I concentrations in the normal age-adjusted reference range, the prevalence of (components of) the MetS did not change after 2 or 5 years, even 10 years of treatment with rhGH [22, 23]. In a study of Profka and his colleagues, they compare both short- (1 year) and long-term (5 years) effects of rhGH in 38 GHD adult patients [19 operated for craniopharyngioma (CP) and 19 for non-functioning pituitary adenoma (NFPA)]. The study suggests that CP patients are less sensitive to the positive rhGH effects on lipid profile and body composition and more prone to insulin sensitivity worsening than NFPA patients, resulting in increased prevalence of MetS in CP only [24]. So there is growing interest in finding an easy and practical tool to using in population studies and clinical practice for evaluating MetS in AGHD. Better risk assessment algorithms are needed to quantify MetS in AGHD. More effectively therapeutic strategies were needed to reverse or delay progression of MetS in AGHD, thereby minimizing problems of increasing cardiovascular disease in this population.

AGHD with MetS is associated with abdominal obesity, blood lipid disorders, insulin resistance, and increased risk of developing cardiovascular disease. Of those, abdominal obesity—the most prevalent manifestation of metabolic syndrome—is a marker of dysfunctional adipose tissue, and is of central importance in clinical diagnosis [25]. Compared with amount of total body fat, the subgroup of individuals with a selective excess of intra-abdominal, or visceral adipose tissue is substantially higher risk of being characterized by insulin resistance and by the features of metabolic syndrome [26, 27]. The VAI, based on simple anthropometric and metabolic parameters, as a surrogate marker of adipose tissue function and distribution independently correlated with cardio-metabolic risk in the general population [10]. VAI showed a strong association with both insulin sensitivity (evaluated with a euglycemic–hyperinsulinemic clamp) and visceral adipose tissue (measured with magnetic resonance imaging) [10]. Although VAI is associated with visceral adipose tissue and cardio-metabolic risk, and a strong relationship among GH axis, VAI and cardio-metabolic risk has been demonstrated in study of Di Somma, an important question has been raised whether VAI is a causal factor or a simple marker of MetS in AGHD. Meanwhile, BMI, WC, WHR, WHtR have been reported as similarly predictive for the presence of MetS in Peruvian adults [20]. The result may be different due to degree and the prevalence varies on the basis of ethnicity, genetic susceptibility and geographic location [23]. So, we anlalyse VAI, BMI, WC, WHR, WHtR with MetS components in Chinese AGHD. We observed WC, BMI, and WHtR were significantly correlated with all MetS components among AGHD. Despite the significant association of VAI with DBP, FPG (*p* < 0.01) in females and DBP, FPG (*p* < 0.05) in males, there was a significant correlation between VAI and MetS components except SBP before and after age and gender adjustment. It is no surprise for association between VAI and TG or HDL, because TG and HDL are used to calculate VAI, in addition to find WHR was only significantly associated with SBP, and FPG. To an extent, the results illustrated all of the adiposity indices was associated with MetS components although the WHR was the weakest one. Our results are generally consistent with some, though not all, prior studies [20, 28, 29]. Insulin resistance is the key etiologic defect that defines metabolic syndrome [30], we find all adiposity indices interested were associated with with HOMA-IR. When we adjust age and gender, the association between VAI and HOMA-IR also exists. According to recently defined criteria, the metabolic syndrome is prevalent and associated with a greater risk of atherosclerotic cardiovascular disease than any of its individual components [29]. In Popa et al.’s study, CKD prevalence and Framingham 10-year CVD risk score were higher in participants with unhealthy metabolic profile [31]. So, we further compared the ability of adiposity indices to predict MetS, and to determine optimal cutoff values for these indices in the diagnoses of MetS among AGHD. We investigated the ROC curve of multiple adiposity measures in predicting MetS among AGHD. Clearly in Fig. 1, the areas under the ROC curve of all adiposity indices were as follows: BMI = 0.812; WC = 0.888; WHR = 0.706; WHtR = 0.902; VAI = 0.920. In addition, ROC curve showed the optimal cutoff values for different adiposity markers in predicting MetS were as follows: WC = 79.65 cm, BMI = 23.46 kg/m^2^; WHR: 0.89 cm/cm; WHtR: 0.54 cm/cm; VAI: 2.29. It could be suggested that BMI, WC, WHR, WHtR and VAI are effective indices with efficacy, cost effectiveness and simplicity of use to prediction incidence of MetS in AGHD. However, the study showed that the best cutoff of WC in predicting MetS in GHD patients was 79.65 cm, i.e., 80 cm. This is a consequence of the definition of MetS, because one of the criteria is to have a WC >80 cm. The ROC analysis on WC, as a consequence, is useless due to collinearity. In conclusion, first, the study illustrated VAI was significant between AGHD with MetS group and Non-MetS group. Second, VAI is the highest area under the ROC curve. Taken together, study indicated VAI was much superior to anthropometric indicators of obesity among BMI, WHR and WHtR for identifying MetS in patients of AGHD.

As yet, there are some limitations in our study that require emphasis. First, because of such a small number of study patients further research should be undertaken in larger sample sizes. Second, the composition of the population studied also impacts largely on the prevalence of differences in ethnicity. As noted by Paniagua et al. [32], heterogeneity in study findings across studies that have assessed cardio-metabolic risk factors in relation to indices of adiposity may be attributable to differences in race/ethnicity, age, and gender distributions of participants across study populations. We only study Asian, thus, caution should be considered when extrapolating our results to other ethnic groups. What is more, this time, we did not take into account the well-known effects of GH on metabolic parameters, and evaluate the effect of the rhGH replacement therapy in these patients. We will study this aspect in future. Finally, we did not analyze the number of other pituitary deficits, which is to better understand if the duration of hypopituitarism can influence. In a word, further research should be undertaken.

In conclusion, the high prevalence of MetS in AGHD, which is increased cardiovascular risk factors, emphasized the importance of using simple, useful, broadly applicable measures in epidemiologic studies to assess MetS of AGHD. Sensitivity of all adiposity indices studied regarding MetS underscores the importance of body fat distribution in determining overall MetS risk in adult growth hormone deficiency. Although, WC, BMI, WHR, WHtR, VAI all present themsefves as easy and practical tools for use in population studies and clinical practice to evaluate MetS in AGHD, in our population-based study, we demonstrated that the VAI ≥2.29 is an indicator (sensitivity and specificity of 98 and 71%, respectively) to best predict MetS in Chinese patients with AGHD.
